# Temporal organization in living systems: toward a physics of life

**DOI:** 10.1140/epjs/s11734-026-02588-1

**Published:** 2026-10-06

**Authors:** Aneta Stefanovska

**Affiliations:** 1 School of Physics and Astronomy, Lancaster University, Lancaster, UK; 2 Department of Systems and Control Engineering, Institute of Science Tokyo, Tokyo, Japan; 3 Faculty of Electrical Engineering, University of Ljubljana, Ljubljana, Slovenia

## Abstract

Classical dynamical systems theory is based on asymptotic stability assumptions. This approach has long dominated theoretical biology, providing insight into stability and functional organization. Yet, living systems rarely converge to static attractors; rather, they adapt continuously and exhibit structural plasticity. We examine the limitations of purely asymptotic descriptions and consider extensions such as metastability and chronotaxicity. We argue that temporal organization provides a useful perspective for understanding living systems and examine how chronotaxicity offers a mathematical description of systems where function and stability can emerge from the maintenance of timing relationships across scales through mutual coupling. This synthesis points toward the development of a broader physics of living systems that integrates non-equilibrium and non-autonomous dynamics with measurable coordination invariants, while providing a basis for both theoretical formalization and the extraction of biologically relevant information from time-series data.

## Introduction

The study of living systems has traditionally relied on asymptotic dynamical descriptions, which characterize biological behavior through long-term patterns such as fixed points or recurring cycles. These approaches have provided important insights across physiology, neuroscience, systems biology, and ecology, offering valuable tools for the analysis of oscillations and networks [30, 52, 61, 85, 126, 140].

Yet, despite their success, such descriptions can face limitations when applied to systems whose behavior is shaped by continuous adaptation, changing interaction structures, and evolving relationships across multiple temporal scales. Living systems maintain function while undergoing structural changes, environmental fluctuations, and variations in internal coordination. Their organization is therefore expressed not only through stationary states, but also through the preservation of temporal relationships among interacting components and processes. In such systems, coordination is maintained through ongoing dynamical interactions rather than solely through convergence toward fixed states.

Descriptions focused primarily on state variables and asymptotic behavior may therefore overlook temporal organization that contributes to functional coordination in biological systems. This motivates a process-centric perspective, in which coordination emerges from evolving interactions rather than from static state structures.

Recent theoretical advances, including work on phase-based brain metabolism [12], have further emphasized the role of temporal relationships and phase coordination between subsystems. These developments align with emerging concepts in metastability [38, 54, 135], relational phase networks [13, 60, 98], and chronotaxic dynamics [127–129], which collectively suggest that function and stability in living systems may involve persistent temporal organization rather than arising exclusively from fixed asymptotic states.

Chronotaxicity is formulated within the broader theory of non-autonomous dynamical systems. It describes a class of systems in which stability is maintained relative to evolving attracting structures, extending classical notions of attraction and stability to situations where the relevant dynamical organization changes in time. Its relevance to living systems lies in providing a mathematical description of temporal organization under continuous change, complementing established approaches based on autonomous dynamics, stochastic processes, and synchronization.

In this review, we synthesize these approaches within a physics-informed description of biological complexity. We highlight the limitations of purely asymptotic descriptions while emphasizing how non-autonomous and temporally structured dynamics extend classical methods in a natural way. We then examine how temporal coordination, finite-time stability, and multiscale organization emerge from interacting dynamical processes, and review experimental and modeling studies spanning neural, cardiovascular, metabolic, and circadian systems. This synthesis aims to provide a conceptual and mathematical basis for understanding how living systems may exhibit organization through time-dependent relational structures in addition to classical descriptions based on asymptotic states and invariant structures.

## Classical asymptotic approaches to dynamical systems

Theoretical modeling of living systems has traditionally relied on two complementary approaches: deterministic dynamical systems and statistical descriptions based on stochastic dynamics [33, 102, 126]. Within statistical physics, stochastic dynamics provides a mathematical description of systems whose microscopic complexity is represented probabilistically. In their classical formulations, both approaches make extensive use of asymptotic concepts, such as long-time limits, invariant sets, ergodicity, and stationary statistics. The roles of ergodicity and stationarity, together with the relation between time and ensemble averages, are discussed in Sects. 2.3 and 2.4.

### Deterministic autonomous dynamical systems

Living systems are often modeled using deterministic dynamical equations [126]. A general representation for a spatially extended system is

1 $$\begin{aligned} \frac{\partial \textbf{x}(\textbf{r},t)}{\partial t} = \textbf{F}[\textbf{x}(\textbf{r},t),t], \end{aligned}$$

where $\textbf{x}(\textbf{r},t)$ denotes the system state (e.g., concentrations, membrane potentials, densities, or flows), and $\textbf{F}$ is a general nonlinear operator describing the system dynamics and interactions. In the absence of explicit time dependence, $\textbf{F}$ depends only on the state variables and the system is termed autonomous

2 $$\begin{aligned} \frac{d\textbf{x}}{dt}=\textbf{F}(\textbf{x}). \end{aligned}$$

Autonomous systems have formed the foundation of classical dynamical systems theory, where invariant sets and asymptotic stability can be rigorously defined.

Dynamical systems analysis has been developed most comprehensively for autonomous systems, where invariant sets and asymptotic stability can be consistently defined. The mathematical theory of autonomous systems is more complete and classical [126], whereas nonautonomous systems generally require explicitly time-dependent notions of stability, attractors, and invariant structure [6, 56]. Two important special cases arise depending on whether spatial dependence is included:

- *Ordinary differential equations (ODEs):* the state variable depends only on time, $\textbf{x}(t)$, and classical attractor analysis applies.
- *Partial differential equations (PDEs):* spatial dependence is included, allowing the description of diffusion, wave propagation, or spatially coupled oscillators. In this case, asymptotic concepts extend to stationary patterns, limit cycles, or propagating fronts.

The deterministic autonomous dynamical systems share several characteristic features:

1. The theoretical analysis is typically based on asymptotic behavior.
2. The primary focus is on long-time dynamics ($t \rightarrow \infty$), while transient behavior is often treated as secondary.
3. Stability is usually analyzed with respect to fixed points, limit cycles, or stationary and traveling spatial patterns. One important characterization of stability and sensitivity to perturbations uses *Lyapunov exponents*, denoted $\lambda _1 \ge \lambda _2 \ge \dots \ge \lambda _n$, which quantify the average exponential rates of divergence or convergence of nearby trajectories [99]. For deterministic autonomous systems, Lyapunov exponents are defined through the linearized evolution of infinitesimal perturbations. Formally, for an *n*-dimensional system $\dot{\textbf{x}} = \textbf{F}(\textbf{x})$, consider an infinitesimal perturbation $\delta \textbf{x}(0)$. Its evolution is governed by the linearized dynamics
  3 $$\begin{aligned} \dot{\delta \textbf{x}} = D\textbf{F}(\textbf{x}(t)) \, \delta \textbf{x}, \end{aligned}$$
  where $D\textbf{F}(\textbf{x}(t))$ is the Jacobian of $\textbf{F}$ along the trajectory. The Lyapunov spectrum, $\{\lambda _1, \lambda _2, \dots , \lambda _n\}$, is associated with a set of characteristic Lyapunov directions spanning the tangent space along the trajectory. The *i*-th Lyapunov exponent is defined as the asymptotic growth rate of infinitesimal perturbations evolving along the corresponding Lyapunov direction
  4 $$\begin{aligned} \lambda _i = \lim _{t \rightarrow \infty } \frac{1}{t} \ln \frac{\Vert \delta \textbf{x}_i(t)\Vert }{\Vert \delta \textbf{x}_i(0)\Vert }. \end{aligned}$$
  This spectrum characterizes the asymptotic stability properties of the system: positive exponents indicate exponential divergence (chaotic behavior), negative exponents indicate exponential convergence, and zero corresponds to neutrally stable directions. Finite-time Lyapunov exponents (FTLEs) provide a finite-time generalization of this concept, characterizing local exponential growth rates over finite observation intervals rather than in the asymptotic limit. Such finite-time growth rates are not, in general, invariant under changes of norm or coordinate representation and may depend on the chosen phase-space reconstruction, particularly for short observation times [1, 39, 40].
4. Long-time deterministic dynamics is commonly characterized through ergodicity (Sect. 2.3), whereby time averages computed along sufficiently long trajectories are assumed to represent the typical behavior of the system [138].

The mathematical assumption that a system can be described by an autonomous differential equation corresponds physically to assuming either:

- that the system can be treated as effectively isolated from its environment, or
- that interactions with the environment can be approximated as stationary forcing or dissipation.

Many living systems are significantly influenced by time-evolving environmental and physiological conditions, limiting the applicability of purely autonomous descriptions.

Recent work has further emphasized that in explicitly time-dependent dynamical systems, classical notions such as attractors and asymptotic stability may lose their standard interpretation due to the intrinsic nonstationarity of the governing equations [87]. This motivates finite-time approaches to dynamical systems, in which behavior is analyzed on a time-localized basis [56, 82] rather than in the asymptotic limit (see below and in particular Sect. 4).

### Stochastic processes

Whereas deterministic dynamical systems describe the evolution of individual trajectories, statistical physics characterizes systems through probability distributions over ensembles of microscopic states. In practice, these statistical descriptions are frequently formulated using stochastic differential equations or their associated Fokker–Planck equations, where unresolved microscopic degrees of freedom are represented by random processes.

Living systems often exhibit fluctuations arising from intrinsic variability or external influences. Within statistical physics, stochastic dynamics provides a mathematical description of such systems in terms of evolving probability distributions rather than individual trajectories [102]. As in deterministic dynamical systems formulations, the system state may depend on spatial coordinates $\textbf{r}$ and time *t*, denoted $\textbf{x}(\textbf{r},t)$. The dynamics can be represented either by stochastic differential equations describing individual realizations or, equivalently, by the associated Fokker–Planck equation governing the evolution of the probability density functional $\rho [\textbf{x}(\textbf{r}),t]$.

As in deterministic dynamical systems, stochastic systems are termed *autonomous* when their drift and diffusion coefficients are independent of time [6]. Explicit time dependence in the drift, diffusion, or external forcing gives rise to nonautonomous stochastic dynamics [56].

Two commonly used probabilistic descriptions arise in this context. One description is based on the long-time occupation fraction of a trajectory within a region *A* of phase space

5 $$\begin{aligned} P_T(A) = \frac{1}{T} \int _0^T \mathbb {I}\big (\textbf{x}(\textbf{r}, t) \in A\big )\, dt, \end{aligned}$$

where $\mathbb {I}(\cdot )$ equals 1 if its argument is true and 0 otherwise. In the long-time limit, and under appropriate ergodic and stationary conditions, this quantity converges to the corresponding ensemble probability.

A complementary description is based on ensemble averages over many realizations (or replicas) of the system

6 $$\begin{aligned} P(A,t) = \int _{\textbf{x}(\textbf{r}) \in A} \rho [\textbf{x}(\textbf{r}), t] \, \mathcal {D}\textbf{x}(\textbf{r}), \end{aligned}$$

where $\rho [\textbf{x}(\textbf{r}), t]$ denotes the probability density functional over the space of field configurations [102].

In classical statistical–mechanical formulations, several common asymptotic assumptions are often introduced:

1. *Asymptotic limits:* probabilities and averages are defined in the limit of long observation times or large ensembles.
2. *Ergodicity:* long-time averages are assumed to coincide with ensemble averages.
3. *Stationarity:* statistical properties are assumed to be invariant under time translation over sufficiently long intervals.
4. *Thermodynamic description:* macroscopic quantities, such as entropy or free energy, are defined through these asymptotic statistical properties.

Like deterministic dynamical systems, classical stochastic descriptions rely primarily on asymptotic concepts such as ergodicity and stationarity. Recent developments extend these ideas to non-stationary and finite-time settings, which are discussed in the following sections.

### Ergodicity

Ergodicity characterizes the situation when long-time behavior along individual trajectories can be related to statistical properties of the system. We consider the system state as a point $\textbf{x}(t)$ in phase space *X*, which may be finite- or infinite-dimensional depending on whether spatial degrees of freedom are included. We assume that $\textbf{x}(t)$ is generated by a measure-preserving dynamics on (X,μ). Let $A(\textbf{x})$ be an observable defined on *X*.

The *time average* along a trajectory $\textbf{x}(t)$ is

7 $$\begin{aligned} \overline{A}_T = \frac{1}{T} \int _0^T A(\textbf{x}(t)) \, dt, \end{aligned}$$

and the phase-space average with respect to a probability measure μ is

8 $$\begin{aligned} \langle A \rangle = \int _X A(\textbf{x}) \, d\mu (\textbf{x}). \end{aligned}$$

A system is ergodic with respect to μ [53, 138] if for all $\textbf{x}(0)$ outside a set N⊂X with μ(N)=0

9 $$\begin{aligned} \lim _{T \rightarrow \infty } \overline{A}_T = \langle A \rangle . \end{aligned}$$

Ergodicity is closely related to stationarity (Sect. 2.4): stationarity ensures time-translation invariance of statistical properties, while ergodicity additionally concerns equivalence between time and phase-space averages. In living systems, which are often non-stationary and observed over finite-time windows, the ergodic assumption may fail or hold only approximately. In such cases, observables and coordination patterns are often analyzed locally in time, motivating finite-time approaches and the chronotaxic description discussed in Sect. 4.

**Table 1:** Comparison between two classical asymptotic approaches used in the modeling of dynamical systems

Feature	Deterministic dynamical systems	Stochastic, statistical descriptions
Mathematical formulation	Differential equations for system trajectories	Probability distributions and stochastic processes
Mathematical objects	Trajectories, invariant sets, attractors	Probability measures, ensembles, statistical observables
Primary focus	Long-time trajectory behavior ($t \rightarrow \infty$)	Time or ensemble averages in asymptotic limits
Role of fluctuations	Treated as perturbations or external forcing	Incorporated directly into probabilistic description
Ergodicity	Sometimes assumed in statistical interpretations of long-time behavior	Required when relating time and phase-space averages under stationarity
Applicability to living systems	Limited treatment of strong non-stationarity	Typically assumes stationarity and asymptotic limits

### Stationarity and nonstationarity

Classical formulations of ergodicity are developed within the setting of stationary or invariant measures, under which time averages and phase-space (or ensemble) averages can be meaningfully related. Formally, a stochastic process *X*(*t*) is stationary if, for any $n \in \mathbb {N}$, any collection of times $t_1,\dots ,t_n$, and any time shift τ, the joint probability distributions of

10 $$\begin{aligned} (X(t_1),\dots ,X(t_n)) \quad \text {and} \quad (X(t_1+\tau ),\dots ,X(t_n+\tau )) \end{aligned}$$

are identical [27]. For second-order processes, this implies time-independent mean, variance, and autocorrelation functions [100].

Nonstationarity arises when these properties evolve over time, either due to intrinsic dynamics or external influences. In stochastic systems, this may manifest as time-dependent means, variances, or correlations.

Non-autonomous dynamical systems are intrinsically nonstationary in the sense that their governing equations depend explicitly on time, breaking time-translation invariance of the dynamics [56].

In oscillatory systems, nonstationarity can appear even deterministically, through slow variations in amplitude, frequency, or both, causing trajectories to deviate from fixed attractors or limit cycles. Classical statistical tools such as power spectra and autocorrelation functions are based on stationarity assumptions and may therefore misclassify deterministic but time-dependent oscillatory dynamics as stochastic fluctuations [104]. This further motivates the development of methods for the analysis of time-localized finite-time dynamics.

Nonstationary dynamics is therefore common in living systems, where internal regulation and external stimuli continuously modulate the dynamics, challenging the direct application of ergodic or stationary assumptions.

Of special interest is the case of frequency-modulated oscillations, where deterministic non-autonomous driving can significantly modify stability properties. In such systems, a slowly varying frequency can lead to intermittent synchronization regimes, in which trajectories alternate between intervals of local stability and neutral stability. This mechanism has been shown to effectively enlarge synchronization regions (e.g., Arnold tongues) and induce stabilization in parameter regions where the dynamics is otherwise unstable [69, 70].

These effects are particularly relevant in the context of biological oscillators and chronotaxic systems (Sect. 4), where time-dependent modulation of intrinsic frequencies and coupling strengths plays a functional role in maintaining coordination under continuously varying environmental and physiological conditions.

Table 1 summarizes the characteristics of deterministic autonomous dynamical systems and stochastic dynamics as formulated within statistical physics.

## Approaches mitigating asymptotic limitations

Several methods have been proposed to overcome the limitations of classical asymptotic approaches, particularly the reliance on infinite-time assumptions and ergodicity. While these methods partially address non-stationarity and finite-time dynamics, they do not provide a fully unified description.

### Existing partial solutions

#### Non-autonomous dynamical systems

Introducing explicit time dependence in the vector field leads to nonautonomous dynamics and allows for time-dependent attractors, including pullback attractors [56]

11 $$\begin{aligned} \frac{d\textbf{x}}{dt} = \textbf{F}(\textbf{x},t), \quad \frac{\partial \textbf{x}(\textbf{r},t)}{\partial t} = \textbf{F}[\textbf{x}(\textbf{r},t),t] + \boldsymbol{\eta }(\textbf{r},t). \end{aligned}$$

Here, $\textbf{x}(t) \in \mathbb {R}^n$ denotes the state vector of an ODE system, representing quantities such as concentrations, membrane potentials, or population activities, while $\textbf{x}(\textbf{r},t)$ denotes a spatially extended field defined over position $\textbf{r}$ and time *t*. The vector field $\textbf{F}(\textbf{x},t)$ or functional $\textbf{F}[\textbf{x}(\textbf{r},t),t]$ describes the instantaneous system dynamics and depends explicitly on time, capturing the effects of external forcing or slow modulation. The term $\boldsymbol{\eta }(\textbf{r},t)$ represents stochastic influences or fluctuations. The spatial coordinate $\textbf{r}$ is introduced when describing spatially extended or PDE-based models.

This formulation enables the description of non-stationary dynamics through moving or deforming attractors, while asymptotic analysis is applied locally over finite-time windows rather than assuming infinite-time convergence.

#### Finite-time dynamical analysis

Methods such as finite-time Lyapunov exponents (FTLE) and finite-time invariant sets are commonly used to characterize short-term stability and organizing structures in dynamical systems [39, 99, 110]. In particular, they:

- Quantify local rates of expansion or contraction over finite-time intervals;
- Reveal temporally localized attractors, repellers, and transport barriers.

The strength of this approach is that it captures transient organization without requiring ergodicity. However, these results depend on the chosen time window and provide trajectory-dependent characterisations of stability rather than global invariants.

#### Time-localized probabilistic approaches

The stochastic dynamics approach, traditionally formulated in terms of probability distributions and ensemble averages, can also be extended to time-localized or sliding-window distributions [8, 49, 95]. In this setting, statistical properties are characterized over finite temporal intervals, allowing the description of systems with evolving statistical behavior rather than assuming time-independent distributions. One considers:

- Instantaneous correlations, dynamic functional connectivity, or local entropy measures;
- Probabilities estimated over finite-time intervals rather than in the asymptotic limit.

With these modifications, it captures evolving patterns and non-stationary behavior. However, it is typically descriptive and lacks predictive dynamical invariants.

#### Multiscale and hierarchical modeling

Coupled models of fast and slow dynamics (e.g., neuronal activity and metabolism) allow interactions across temporal scales. They can:

- Explicitly separate fast and slow subsystems;
- Reproduce emergent behaviors not visible in single-scale models.

Conceptually introduced by Haken [38] through the *slaving principle*, this approach posits that long-lived (slow) *order parameters* govern the macro-state of a system by enslaving short-lived (fast) microscopic fluctuations. While this approach captures cross-scale interactions, its applicability depends on the presence of a sufficiently clear separation of time scales, which can be affected by coupling between fast and slow dynamics.

Consequently, such models may struggle to characterize moving attractors or define robust finite-time invariants in non-stationary regimes.

### Metastability as a partial relaxation of the asymptotic paradigm

One important development in nonlinear dynamics and theoretical neuroscience aimed at overcoming strict asymptotic convergence is the concept of *metastability* [41, 54]. Metastable systems exhibit long-lived transient states that resemble attractors over finite-time intervals but are not asymptotically stable in the classical sense.

In deterministic dynamical systems, metastability typically arises near weakly unstable invariant sets, where trajectories remain for extended but finite times before escaping. In stochastic systems, it is often characterized by rare transitions between potential wells, with escape times described by classical reaction-rate theory [59] or large deviation theory [32]. In statistical physics, this leads to descriptions in terms of quasi-stationary distributions and exponentially long escape times, with rigorous characterizations often derived in asymptotic limits of small noise or large system size [32, 59].

Although metastable states allow for long-lived transient organization, they remain embedded within the asymptotic description: the underlying structure is still defined relative to invariant sets, energy landscapes, or stationary probability measures, and characterization relies on long-time or ensemble properties.

In theoretical neuroscience, metastability has been proposed as a mechanism for flexible coordination and transient network configurations. Models of large-scale brain activity demonstrate switching among quasi-stable patterns of synchronization, often interpreted as metastable states [26, 50, 54]. However, even in these cases, stability is defined with respect to quasi-stationary states and asymptotic escape times, rather than continuously evolving time-dependent attractors.

For living systems that maintain organization under ongoing change, the relevant structures may not be fixed invariant sets or quasi-stable basins, but rather time-dependent attracting states, trajectories, or manifolds. A more general description, chronotaxic dynamics, formalizes stability with respect to such moving attractors; this description will be introduced in Sect. 4.

Thus, metastability mitigates some limitations of strict asymptotic convergence by capturing long-lived transients and flexible coordination, but it does not fully resolve the conceptual tension between asymptotic invariance and the continuous nonstationarity observed in living systems [41].

**Table 2:** Approaches partially mitigating the asymptotic limitations of classical dynamical descriptions

Approach	Strengths	Limitations
Non-autonomous systems	Allow explicitly time-dependent vector fields and moving attractors	Often rely on prescribed forcing; analysis remains partly asymptotic
Finite-time analysis	Quantifies transient stability and short-term organization	Results depend on chosen time window; characterization is generally local in time
Time-local statistics	Captures evolving correlations and non-stationary behavior	Primarily descriptive; lacks dynamical structure
Multiscale modeling	Represents interactions across temporal scales	Requires scale separation; does not ensure moving invariant structures
Metastability	Long-lived transient states; flexible switching	Stability defined relative to fixed landscape; asymptotic escape times

### From metastability to time-dependent invariance

The approaches discussed in Sects. 3.1 and 3.2, and summarized in Table 2, partially relax asymptotic assumptions but do not provide a unified formulation of finite-time, locally invariant dynamics.

Classical dynamical descriptions have been refined in several directions: non-autonomous formulations introduce time-dependent vector fields, finite-time analysis quantifies transient stability, and metastability captures long-lived yet ultimately transient states. However, organization is still defined relative to invariant or quasi-invariant structures characterized through asymptotic behavior.

In living systems, organization often persists under continuous change. Physiological regulation, cell signaling, metabolic coordination, and brain dynamics exhibit sustained attraction to structures that themselves evolve, so that the reference configuration is inherently time-dependent. Similar behavior appears in gene regulation and development, where function is maintained despite ongoing turnover and environmental fluctuations. These systems operate far from equilibrium, sustained by continuous fluxes of energy and matter, and display robustness without relying on static stability.

This observation motivates a conceptual shift: stability can be defined relative to moving attracting trajectories or manifolds. Organization is then maintained locally in time, and classical invariance is replaced by dynamically evolving structures. *Chronotaxic systems*, introduced below, formalize this idea by providing a formal description of organized dynamics without relying on asymptotic stationarity.

## Chronotaxic systems: finite-time stability and moving attractors

Following these developments, *chronotaxic systems* (from *chronos* – time, and *taxis* – order in Greek) were introduced by Suprunenko *et al*. [127–129] to describe a specific form of organization in non-autonomous dynamical systems. Chronotaxic systems are characterized by a stable moving point attractor that evolves continuously in time while maintaining local dynamical organization. They can therefore be viewed as a class of non-autonomous dissipative systems in which attraction occurs toward an evolving trajectory rather than toward a stationary invariant structure.

The mathematical description of chronotaxic systems is naturally related to the theory of non-autonomous dynamical systems, including cocycles, pullback attraction, and time-dependent attracting structures [6, 7, 56]. However, chronotaxicity represents a more specific dynamical property than the existence of a general pullback or random attractor: it emphasizes the persistence of a stable moving point attractor and the associated local contraction of nearby trajectories [127–129]. In this sense, chronotaxicity is related to, but distinct from, broader concepts of pullback and random attractors, forward–pullback dynamics, statistical attractors, and tracking phenomena in non-autonomous systems [86].

The stability of chronotaxic organization is defined locally in time through instantaneous contraction toward the moving attractor. This can be characterized using local finite-time stability measures, such as the local Lyapunov exponent $\lambda _{\max }(t)$, with negative values indicating contraction within the time-dependent attracting region. Unlike classical asymptotic stability, which characterizes convergence in the limit $t\rightarrow \infty$, chronotaxic stability concerns the maintenance of organization under continuously evolving dynamical conditions. This provides a mathematical description of systems that preserve coordinated behavior while their characteristic frequencies, parameters, or interaction patterns change over time.

### Definition and core principles

*Cyclic processes*, also known as *oscillators*, are ubiquitous across all scales, e.g., the beating of the heart, respiration, neuronal activity, and planetary orbits. Positions along the cycle are parameterized by an angular variable $\theta \in [0,2\pi )$, referred to as the *phase*. The phase description employed here is consistent with standard phase-reduction approaches for nonlinear oscillators [81]. For oscillators with constant natural frequency *f*, the phase θ is defined, such that

$$\dot{\theta }(t) = 2\pi f,$$

where $\dot{\theta }(t)$ is the instantaneous angular velocity. More generally, under slow modulation of the instantaneous frequency *f*(*t*), the phase dynamics satisfies

$$\dot{\theta }(t) \approx 2\pi f(t),$$

where ≈ indicates that higher-order corrections are neglected under a separation-of-timescales assumption [82, 83].

An *oscillator network* is a collection of interacting oscillators. *Synchronization* in such a network refers to the phenomenon where interacting oscillators with different natural frequencies evolve with a common asymptotic frequency (frequency locking). The region in parameter space where a periodically forced nonlinear system becomes frequency-locked (synchronized) to the forcing is known as an *Arnold tongue* [98]. These regions typically have a tongue-like shape when plotted, showing where stable synchronization occurs. To formalize these ideas, consider a nonautonomous dynamical system describing *N* coupled oscillators

12 $$\begin{aligned} \frac{d\textbf{x}}{dt} = \textbf{F}[\textbf{x},t], \quad \textbf{x}(t) = (\theta _1(t), \dots , \theta _N(t))^T, \end{aligned}$$

where $\theta _j(t)$ denotes the phase of oscillator *j*. The system is chronotaxic if there exists a reference trajectory $\textbf{x}_A(t)$, such that:

1. $\textbf{x}_A(t)$ is a solution of the system: $\frac{d\textbf{x}_A}{dt} = \textbf{F}[\textbf{x}_A,t]$;
2. There exists a time-dependent contraction region $C(t)\subset \mathbb {R}^N$ surrounding $\textbf{x}_A(t)$;
3. For all $\textbf{x}(t_0) \in C(t_0)$, trajectories remain within the contracting region and converge toward the reference trajectory $\textbf{x}_A(t)$, that is
  13 $$\begin{aligned} \frac{d}{dt} \Vert \textbf{x}(t) - \textbf{x}_A(t)\Vert < 0 \quad \text {within } C(t), \ \textbf{x}(t)\ne \textbf{x}_A(t). \end{aligned}$$

Equivalently, the largest instantaneous Lyapunov exponent $\lambda _{\max }(t)$ within *C*(*t*) is negative at all times, consistent with the generalized contraction-based formulation of chronotaxic systems [129].

In the idealization that the system has an infinite history over which $\lambda _{\max }(t)$ remains below some negative value, the reference trajectory $\textbf{x}_A(t)$ is uniquely determined by the driving dynamics. In this case, trajectories originating from different initial conditions within the contracting region converge toward the reference trajectory in the pullback sense, namely in the limit as the start time of those trajectories tends to -∞ [127, 129].

When the finite history of the system prior to time *t* is explicitly considered, $\textbf{x}_A(t)$ is not strictly uniquely determined to infinite precision. However, provided that the available history is long compared with the contraction timescale associated with the inverse magnitude of $\lambda _{\max }$, the remaining dependence on the initial conditions becomes negligible for practical purposes. The resulting uncertainty in the trajectory is then much smaller than the differences between experimental observations and any non-overfitted model. For a fixed starting time, trajectories with different initial conditions within the contracting region rapidly become practically indistinguishable, providing a finite-time approximation to the reference trajectory $\textbf{x}_A(t)$. The continuing contraction property then ensures that subsequent perturbations to the driven state $\textbf{x}(t)$ decay over time, so that even in the presence of small ongoing perturbations, the driven state remains close to the evolving trajectory whose time dependence is governed, to high precision, by the driving dynamics.

In this way, a chronotaxic system is a nonautonomous system with a continuously evolving yet locally stable reference trajectory. In the original formulation of chronotaxic systems, this stability is typically associated with a separable structure consisting of a driving subsystem and a driven subsystem with predominantly unidirectional coupling. This ensures that the reference trajectory $\textbf{x}_A(t)$ is determined entirely by the driver and remains unaffected by perturbations to the driven dynamics.

Without this condition, a negative instantaneous Lyapunov exponent merely characterizes synchronization and does not guarantee robustness of the reference trajectory itself. Accordingly, chronotaxicity captures stability relative to an externally defined, time-dependent attractor, rather than stability emerging solely from internal dynamics. This notion of stability is local in time and does not rely on asymptotic limits or ergodic assumptions. Such properties place chronotaxic systems within the broader class of nonautonomous and driven dynamical systems, while emphasizing finite-time stability and continuously evolving attracting trajectories.

MacKay [71] further related chronotaxic dynamics to the theory of normally hyperbolic invariant structures in nonautonomous oscillator systems. In this formulation, the reference trajectory $\textbf{x}_A(t)$ is interpreted as a persistent attracting invariant trajectory generated by the evolving driver, thereby connecting chronotaxicity with the broader theory of forced oscillators and nonautonomous synchronization.

Experimental evidence for related nonautonomous tracking phenomena has recently been provided by Xu, Kuhl, and Noiray [137], who demonstrated dynamic synchronization of a self-sustained aeroacoustic oscillator under slowly varying forcing. In the strict synchronization regime, the oscillator phase follows an evolving drive phase, while amplitude fluctuations are suppressed. Although formulated in terms of dynamic synchronization rather than chronotaxicity, this result illustrates the stability of non-stationary oscillatory states under deterministic time-dependent forcing.

In control theory, this condition corresponds to a tracking stability property with respect to the time-dependent reference trajectory $\textbf{x}_A(t)$. Stability corresponds to the decay of deviations (tracking error) along the flow, rather than convergence to a fixed equilibrium point. The condition $\lambda _{\max }(t) < 0$ is thus interpreted as a local contraction condition ensuring that infinitesimal perturbations to the reference trajectory decay in time [68], consistent with classical Lyapunov analysis of nonlinear tracking systems and disturbance rejection.

This description is closely related to the classical theory of generalized synchronization in unidirectionally coupled dynamical systems. In particular, Kocarev and Parlitz [57] showed that synchronization can be characterized by functional dependence between driver and response systems rather than asymptotic convergence to a fixed invariant set. Chronotaxicity can therefore be interpreted as a time-dependent extension of generalized synchronization, where the synchronizing relationship is continuously evaluated along an evolving reference trajectory. In contrast to random attractors in stochastic dynamical systems and synchronization phenomena induced by common noise or chaotic driving, chronotaxicity explicitly refers to a deterministic, time-dependent reference trajectory that is externally driven and locally contracting, ensuring stability with respect to this evolving reference, rather than to a statistical or ensemble-based notion of stability.

An example illustrating how time-dependent synchronization can emerge in non-autonomous oscillator systems is provided by Petkoski and Stefanovska [97], who extended the Kuramoto model to ensembles with time-dependent natural frequencies $\omega _j(t)$ and couplings $K_{ij}(t)$. In their time-dependent formulation, the collective mean field acts as a moving reference trajectory that guides the ensemble. Their results demonstrate that, even under deterministic temporal modulation, the ensemble remains synchronized, with trajectories continuously attracted toward this evolving reference over finite times. This work provides an early, analytically tractable example of behavior consistent with the later formalization of chronotaxic principles in oscillator networks.

Temporal variability in oscillator properties can also influence collective synchronization. Aravind et al. [5] showed that repeated reshuffling of intrinsic frequencies in Kuramoto-type oscillator networks can enhance synchrony and reduce the coupling required for phase locking. Although this mechanism differs from chronotaxicity, which concerns stability relative to a time-dependent attracting trajectory, it highlights how time-dependent oscillator dynamics can generate robust organization in nonstationary environments. These developments also suggest the need for phase descriptions that explicitly account for time-dependent dynamics, extending beyond concepts originally developed for stationary oscillators.

Taken together, chronotaxicity highlights the importance of temporal organization in sustaining function in living systems, complementing structural and asymptotic descriptions of stability. By stabilizing trajectories over finite times while remaining adaptive and resilient, chronotaxic dynamics provides a mechanistic basis for understanding coordination across scales. This formulation emphasizes function-centric stability, highlighting that biological robustness arises from maintained temporal relations rather than asymptotic equilibrium, and sets the stage for integrating chronotaxicity with broader concepts of nonautonomous, non-ergodic dynamics in living systems. Chronotaxic systems therefore provide a useful mathematical formalism for modeling certain classes of continuously adapting and non-stationary living systems. By formalizing stability over finite times with moving, time-localized attractors, they capture the dynamic, adaptive, resilient, and coordinated behavior observed across multiple scales, linking theoretical descriptions with experimentally measurable oscillatory and relational dynamics.

### Phase representation of chronotaxic oscillators

We consider a phase–amplitude representation of oscillatory dynamics in which the system is described by a dynamical amplitude *r*(*t*) and phase ϕ(t). This representation is understood as a model-dependent coordinate decomposition of an underlying oscillatory system with a dominant rhythmic component.

In these coordinates, the dynamics take the form

$$\begin{aligned} \dot{r}&= F(r,\phi ,t), \\ \dot{\phi }&= \omega (r,\phi ,t), \end{aligned}$$

where *F* and ω are the amplitude and phase components induced by the chosen representation of the full nonautonomous dynamics.

The variable *r*(*t*) represents the dynamical amplitude of the oscillator, which in empirical applications is identified with a reconstructed instantaneous amplitude *A*(*t*) obtained via ridge-based or related time–frequency decomposition methods, under the assumption of a well-defined dominant oscillatory mode [48].

Chronotaxicity requires that both the amplitude and phase dynamics possess mechanisms of local contraction. For the phase dynamics, this mechanism arises entirely from the presence of time-dependent driving dynamics, since autonomous oscillators cannot admit an invariant region of contracting phase dynamics. For the amplitude dynamics, the contraction is intrinsic to the internal oscillator dynamics, but the value toward which the amplitude stabilizes changes over time due to the time-dependent driving dynamics. This scenario is represented by the existence, at each time *t*, of a time-dependent instantaneous equilibrium $r_A(t)$ of the radial dynamics [124, 125, 126], defined implicitly by

$$F(r_A(t),\phi ,t)=0, \qquad \left. \frac{\partial F}{\partial r}\right| _{(r_A(t),\phi ,t)}<0$$

for admissible values of ϕ and *t*.

In the full nonautonomous system, this structure is associated with the existence of a reference trajectory $\textbf{x}_A(t)$ introduced above, as defined in [127–129].

The main dynamical implications can be summarized as follows:

1. *Time-localized stability*: Within the contraction region, perturbations decay and trajectories remain close to the reference trajectory $\textbf{x}_A(t)$ over finite-time intervals. If the attractor evolves too rapidly relative to the system’s intrinsic response time, trajectories may leave the contraction region, leading to loss of chronotaxicity and transient desynchronization.
2. *Continuous adaptation*: The attracting structure evolves in response to internal or external driving.
3. *Finite-time invariance*: Stability is understood locally in time without assuming global asymptotic invariance.
4. *Robustness to perturbations*: Small disturbances decay within the contraction region, maintaining temporal organization.

**Table 3:** Conceptual distinctions between classical asymptotic and chronotaxic descriptions of dynamical systems

Feature	Classical asymptotic descriptions	Chronotaxic dynamics
Attractor	Fixed or stationary (invariant as $t \rightarrow \infty$)	Moving, time-dependent attractor
Temporal organization	Infinite-time limit; asymptotic behavior dominates	Finite-time, locally defined behavior
Stability notion	Global or asymptotic stability	Finite-time stability
Ergodicity assumption	Often assumed	Not required
Relevance to living systems	Limited for strongly non-stationary or adaptive dynamics	Captures adaptive, continuously evolving organization

Table 3 outlines the conceptual differences between classical asymptotic approaches and the chronotaxic description of dynamical systems, focusing on their respective stability concepts and implications for modeling living systems. Classical approaches assume fixed or stationary attractors and emphasize asymptotic behavior in the long-time limit, whereas chronotaxic systems are characterized by moving, time-dependent attractors and stability defined locally in time. Such properties make them suitable for describing adaptive and strongly non-stationary biological processes.

### Positioning chronotaxicity among concepts of temporal organization

**Fig. 1:**
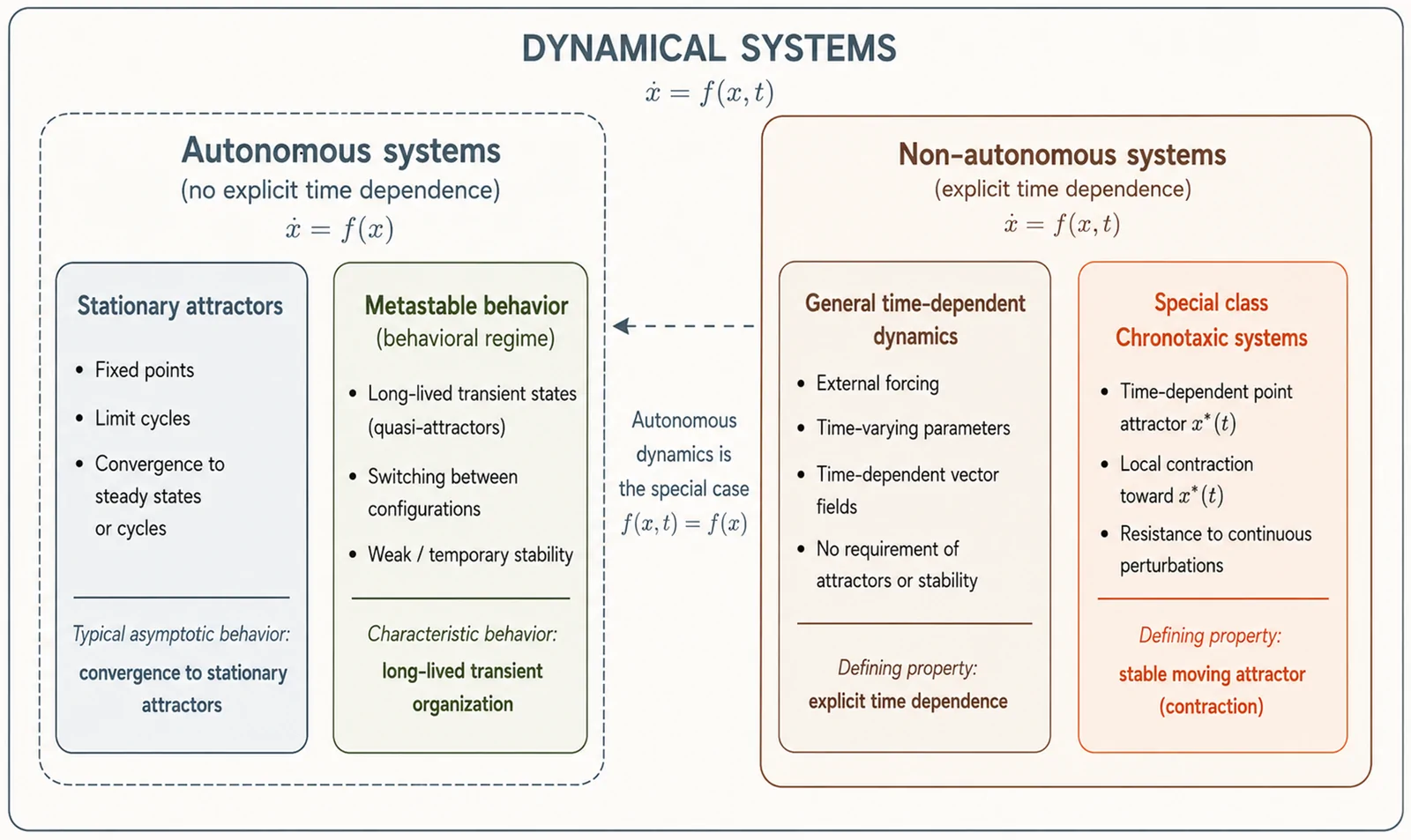
Overview of dynamical systems according to the presence or absence of explicit time dependence. Autonomous systems $\dot{x}=f(x)$ may converge to stationary attractors or exhibit metastable behavior characterized by long-lived transient organization and switching between quasi-stable states. Non-autonomous systems $\dot{x}=f(x,t)$ describe dynamics with explicit temporal dependence and include chronotaxic systems as a specific subclass characterized by a stable moving attractor maintained through local contraction. Deterministic and stochastic formulations can occur within both autonomous and non-autonomous settings

As discussed in the previous sections, dynamical systems theory provides a range of complementary descriptions of organization, stability, and temporal evolution. These descriptions differ according to whether the organizing structures are stationary or evolving, whether organization arises through attraction or coordination, and whether stability is defined through asymptotic convergence or finite-time organization. Figure 1 summarizes the conceptual relationship between autonomous, non-autonomous, and chronotaxic systems.

Chronotaxicity should be viewed within the broader landscape of non-autonomous dynamical systems. Several existing concepts describe different aspects of time-dependent dynamics. The purpose of the following comparison, summarized in Table 4, is therefore not to claim mathematical independence from existing concepts, but to clarify the particular organizational properties emphasized by chronotaxicity.

Stationary attractors provide the classical dynamical description of organization through convergence toward time-independent invariant structures. However, biological systems often exhibit persistent organization without convergence toward a single fixed state. Metastability has been widely used within dynamical systems approaches to describe such behavior, where long-lived transient states and switching between quasi-stable configurations provide a form of organized dynamics without requiring asymptotic convergence [41, 54]. Although initially developed for autonomous systems, metastability concepts have subsequently been extended to systems with explicit temporal variation, external forcing, and interacting components.

Non-autonomous dynamical systems provide a general mathematical setting for situations in which the governing dynamics evolves explicitly in time [6, 7, 56]. Within this setting, forward attractors, pullback attractors, and random attractors describe different forms of time-dependent organization. These approaches demonstrate that evolving dynamical structures can be treated rigorously within established dynamical systems theory. Non-autonomous dynamics is a necessary condition for chronotaxicity, but not a sufficient one. Chronotaxic systems form a particular subclass of non-autonomous systems possessing time-dependent attracting structures that confer finite-time stability despite their continuously evolving dynamics.

Other approaches focus on temporal coordination and trajectory organization rather than attracting structures. Tracking stability describes convergence toward a prescribed time-dependent trajectory, a concept widely used in control theory and trajectory stabilization problems [55], whereas generalized synchronization and entrainment describe coordination arising from coupling or external forcing [57, 98, 140]. Although these approaches address important aspects of temporal organization, they differ from chronotaxicity in their definition of the organizing trajectory or mechanism of coordination.

**Table 4:** Conceptual comparison of dynamical concepts relevant to temporal organization

Dynamical concept	Main feature	Relationship to chronotaxicity
Stationary attractors	Time-independent attracting structures in autonomous systems	Provide the classical stability concept. Chronotaxicity extends attraction to an evolving moving point attractor.
Metastability	Long-lived transient organization and switching between quasi-stable states	Describes organization without convergence to a single attractor. Chronotaxicity instead involves continuous attraction toward an evolving trajectory.
Forward attractors	Future attracting structures in autonomous or non-autonomous systems	Describe attraction in evolving systems but do not necessarily imply a unique moving attracting point.
Pullback attractors	Time-dependent attracting structures determined by system history	Closely related through non-autonomous attraction; chronotaxicity is a more specific case involving a stable moving point attractor.
Random attractors	Attracting structures in stochastic dynamical systems	Extend attraction concepts to random environments; chronotaxicity concerns stable organization of an evolving trajectory.
Tracking stability	Convergence toward a prescribed reference trajectory	Shares trajectory-based stability, but the trajectory in chronotaxicity is an intrinsic property of the dynamics.
Generalized synchronization	Functional coordination between coupled systems	Describes relationships between systems rather than a moving attracting state.
Entrainment	Phase or frequency locking induced by external forcing	Describes externally driven coordination and does not require an intrinsic moving attractor.
Chronotaxicity	Stable moving attracting structure in a non-autonomous system	Characterizes stable organization despite continuously varying dynamical properties; in biological applications often concerns oscillatory systems.

These concepts describe complementary aspects of dynamical organization, including attraction, transient stability, coordination, and time-dependent evolution. Chronotaxicity represents a specific subclass of non-autonomous dynamics characterized by a stable moving point attractor

Chronotaxicity therefore occupies a specific position within this landscape. It describes a subclass of non-autonomous systems characterized by the existence of a stable moving point attractor toward which nearby trajectories continuously contract. In such systems, characteristic frequencies and dynamical properties may vary over time, while stable organization is maintained. Thus, chronotaxicity does not replace existing concepts in non-autonomous dynamics, synchronization theory, or stochastic systems, but identifies a specific dynamical property of systems in which the organizing trajectory itself evolves in time. Chronotaxicity was introduced not because existing non-autonomous dynamical systems theory was mathematically insufficient, but because biological systems motivated a description in which organization is maintained relative to evolving attracting structures whose stability properties can be quantified experimentally.

This perspective is particularly relevant for biological systems, where physiological and neural processes often exhibit continuously varying frequencies, adaptive coupling, and coordination across multiple temporal scales. An important methodological challenge concerns the reconstruction of the underlying time-dependent dynamics from empirical observations. Once the relevant dynamical variables and reference trajectory are obtained, the defining properties of chronotaxicity, including attraction toward the evolving trajectory and local contraction, can be directly assessed, as discussed in Sect. 4.4. In common with other dynamical concepts inferred from experimental data, such identification depends on the available observables, reconstruction methods, and modeling assumptions.

### Detection of chronotaxicity from data

Experimentally, chronotaxic behavior can be investigated through several dynamical features:

- Persistent phase relationships associated with an evolving attracting trajectory,
- Finite-time convergence toward a moving oscillatory trajectory,
- Absence of sustained drift away from the evolving attractor,
- Robustness under moderate perturbations.

The experimental identification of chronotaxicity requires reconstruction of the underlying time-dependent reference dynamics. Once the reference trajectory is reconstructed, chronotaxicity can be assessed by evaluating whether perturbations remain bounded and contract toward this evolving trajectory [22, 63, 127].

An important methodological advance in reconstructing time-varying dynamical interactions is the dynamical Bayesian inference method developed by Stankovski et al. [116, 119], which enables the estimation of evolving coupling structures directly from data. The method permits reconstruction of time-dependent frequencies, coupling functions, and interaction directionality, thereby allowing externally driven dynamics to be distinguished from mutual synchronization. In chronotaxic systems, these properties enable identification of the underlying driver-driven organization and tracking of local stability properties over time. Dynamical Bayesian inference therefore provides a practical methodology for reconstructing and characterizing the time-dependent dynamical interactions underlying chronotaxic systems.

It should be noted that non-stationary synchronization or frequency modulation alone is not sufficient criterion for chronotaxicity; rather, they provide information required to reconstruct the evolving dynamical organization and test whether it satisfies the defining properties of chronotaxic systems.

Practical criteria for identifying chronotaxicity were proposed by Clemson *et al*. [22]:

1. Reconstruction of the phase space from observed time-series,
2. Estimation of the radial dynamics $\dot{r}$,
3. Verification of persistently negative radial stability,
4. Demonstration of recovery following perturbations.

These criteria were subsequently extended by Lancaster *et al*. [63] to address additional experimental challenges, including

- Detection in the presence of noise,
- Strong external forcing,
- Statistical validation of the inferred dynamics.

Importantly, the defining stability properties of chronotaxic systems can be assessed within finite experimental windows without requiring assumptions of stationarity or ergodicity.

### Mode decomposition as a quantitative tool

To obtain a quantitative description of the moving attractor $x_A(t)$ and its coordination properties, mode decomposition methods provide a natural approach. These techniques aim to represent complex dynamical systems or time-series as sums of simpler components called modes, each capturing coherent patterns in space, time, or frequency. Mode decomposition is widely used in fluid dynamics, neuroscience, cardiovascular science, climate science, and mechanical vibrations, where understanding the structure of high-dimensional or nonlinear data is crucial.

The main approaches can be categorized as linear and nonlinear. Linear methods assume that the system can be represented as a linear superposition of modes. Common examples include Fourier decomposition, the Karhunen-–Loève (K–L) method (also known as proper orthogonal decomposition, POD, [43]), which extracts orthogonal modes that optimally capture variance, and dynamic mode decomposition (DMD), which identifies modes associated with specific temporal frequencies and growth or decay rates (see Sect. 4.5.1). Nonlinear methods, such as nonlinear mode decomposition (NMD), Sect. 4.5.2, explicitly handle amplitude- and frequency-modulated oscillations.

#### Dynamic mode decomposition

DMD [107] is a data-driven technique for extracting coherent spatiotemporal patterns from high-dimensional systems. Given a sequence of system states $\textbf{x}_k$, one can approximate the (generally nonlinear) dynamics $\textbf{F}[\textbf{x}]$ by a linear evolution operator $\textbf{A}$, such that

$$\textbf{x}_{k+1} \approx \textbf{A}\textbf{x}_k.$$

The spectral properties of $\textbf{A}$ yield modes associated with characteristic growth rates and oscillation frequencies.

In practice, DMD is computed via a low-rank decomposition of snapshot data, providing a reduced representation of the dominant dynamical structures. It can be interpreted as a finite-dimensional approximation of the Koopman operator [105], enabling the analysis of nonlinear systems through linear techniques.

In the context of chronotaxic systems, DMD can identify dominant oscillatory components, track slow evolution of time-dependent attractors, and characterize transient dynamics. However, its linear approximation may limit performance in strongly nonlinear or highly nonstationary regimes. The key features are summarized in Table 5.

**Table 5:** Comparison of DMD and NMD highlighting differences in representation, time variation, robustness, interpretability, and applicability to nonstationary systems

Feature	DMD	NMD
Linearity	Linear in observables	Nonlinear representation in amplitude–phase variables (A(t),ϕ(t))
Dimensionality	High-dimensional snapshot data	Low-dimensional modes extracted from signals
Time-variation	Fixed-frequency modes (extensions required)	Explicitly time-varying amplitude and frequency
Noise robustness	Moderate; improved via SVD truncation	Enhanced robustness via statistical validation of modes
Physical interpretability	Modes may admit physical interpretation depending on system	Modes correspond to dominant oscillatory components in nonstationary signals
Best suited for	Complex fluids, networked systems	Biological signals, mechanical oscillators, nonstationary time-series
Relevance for chronotaxic systems	Captures dominant oscillatory structures and slow drift of effective modes	Resolves time-localized oscillatory structure and finite-time dynamics

The comparison is qualitative and intended to highlight conceptual differences in typical usage rather than strict methodological boundaries. We note that classical DMD is fundamentally based on a linear approximation of the underlying dynamics. However, several nonlinear extensions have been developed, most notably Extended DMD (EDMD), which lifts the dynamics into a higher-dimensional observable space to approximate the Koopman operator and thereby capture nonlinear behavior more effectively [62, 105, 139].

#### Nonlinear mode decomposition

In non-autonomous oscillatory systems, the identification of dynamical modes requires distinguishing independent oscillatory processes from harmonics of the same underlying oscillation. This distinction is particularly important for systems with time-varying frequencies, where harmonic components no longer appear as fixed spectral peaks but evolve together with the fundamental oscillation. Time-frequency methods combined with statistical testing can identify such harmonic relationships and prevent the misinterpretation of harmonics as independent oscillatory components [111].

NMD explicitly extracts time-varying amplitude and phase components from oscillatory signals [46]

14 $$\begin{aligned} x(t) = \sum _{j=1}^{N} A_j(t) \cos \big (\phi _j(t)\big ) + \eta (t), \end{aligned}$$

where $A_j(t)$ and $\phi _j(t)$ are the instantaneous amplitude and phase of mode *j*, and η(t) represents residual noise.

The NMD procedure involves:

1. *Time-frequency representation*: Continuous wavelet transform or synchrosqueezed transform to map the signal into a time-frequency plane [47].
2. *Ridge extraction*: Dominant oscillatory components are identified by tracing ridges in the time-frequency plane [48].
3. *Amplitude and phase reconstruction:* Instantaneous amplitude $A_j(t)$ and phase $\phi _j(t)$ are reconstructed along each ridge.
4. *Statistical significance testing:* Surrogate data are used to separate meaningful modes from noise [66].

Such a decomposition is particularly well suited for investigating chronotaxic dynamics in biological systems, since it provides adaptive, noise-robust extraction of dynamically evolving amplitude and phase components [46]. The resulting modes are physically interpretable oscillatory components, with harmonics represented as dynamically related components of the same underlying oscillatory process rather than independent modes. The key features of NMD and its differences from DMD are summarized in Table 5.

### Order parameters

Once oscillatory modes have been extracted, their coordination can be quantified using *phase-based* and *angular velocity-based* synchronization measures. Importantly, these measures can be evaluated over *finite-time windows*, providing a non-asymptotic characterization of coordination, which is particularly relevant for non-stationary and adaptive systems.

**Kuramoto order parameter.** For a network of *N* phase oscillators with phases $\phi _j(t) \in [0,2\pi ]$, the Kuramoto mean field [3, 61] is

$$\mu (t) = \frac{1}{N} \sum _{j=1}^N e^{i \phi _j(t)},$$

with magnitude

$$r(t) = |\mu (t)| \in [0,1].$$

Here, r(t)=1 corresponds to complete phase alignment, while r(t)=0 indicates fully incoherent phases. While traditionally interpreted as a long-term (asymptotic) measure, *r*(*t*) can also be computed over finite-time intervals to capture *instantaneous or transient synchronization*, which is essential for analyzing nonautonomous and chronotaxic systems.

**Velocity-alignment order parameter.** To capture *frequency synchronization*, particularly in heterogeneous or time-dependent networks, we define

15 $$\begin{aligned} R(t) = \max \!\Bigg ( 1 - \frac{\textrm{var}(\dot{\phi }_1(t),\dots ,\dot{\phi }_N(t))}{\textrm{var}(\omega _1(t),\dots ,\omega _N(t))}, \, 0 \Bigg ), \end{aligned}$$

where $\dot{\phi }_j(t)$ are instantaneous angular velocities and $\omega _j(t)$ are the internal (uncoupled) frequencies. *R*(*t*) measures the variance reduction induced by coupling, providing a *finite-time, non-asymptotic measure* of coordination [84].

Alternative approaches exist to quantify coordination in oscillator ensembles. For example, the mean-field variability index κ introduced in [113] characterizes synchronization through fluctuations in the ensemble’s overall activity, serving as a complementary macroscopic measure. A related approach is the universal order parameter for finite networks introduced by Schröder et al. [109], which captures partial phase locking and synchronization transitions in networked oscillator systems.

These synchronization metrics allow one to:

- Quantify the *strength of synchronization* (phase or frequency),
- Detect *partial synchronization or clustering*,
- Identify *transitions* between coordinated and desynchronized states,
- Characterize *finite-time stability* of coordination.

When combined with *chronotaxicity*, mode decomposition [46], coupling functions [119], and phase coherence measures [9], *r*(*t*) and *R*(*t*) provide a complementary set of quantitative tools for analyzing moving attractors and adaptive coordination in nonautonomous biological systems, explicitly accounting for non-asymptotic dynamics.

## Case study: heart rate variability

Heart rate variability (HRV) refers to the naturally occurring fluctuations in the interval between consecutive heartbeats, reflecting the respiratory, autonomic nervous system and vascular regulation of the heart rate.

Key contributions in HRV research include the following:

- Hon and Lee (1965) [44]: First clinical recognition of beat-to-beat variability.
- Peňáz et al. (1968) [94]: Early spectral decomposition of cardiovascular rhythms.
- Sayers (1973) [106]: Systematic study of structured HRV components in humans.
- Akselrod et al. (1981) [4]: Standardized spectral metrics; historically foundational but limited in capturing non-stationary oscillations.
- Bračič Lotrič et al. (2000) [20]: Wavelet-based analysis capturing non-stationary HRV oscillations.

This lineage emphasizes the evolution from observation of the HRV phenomenon to methods that faithfully reveal its physiological complexity.

HRV is typically measured as RR intervals from electrocardiogram (ECG) recordings [15, 25, 79, 131] and traditionally analyzed using spectral analysis methods [93]. Alternative approaches for obtaining HRV include photoplethysmography (PPG) or beat-to-beat blood pressure recordings [34]. The pulse rate variability (PRV) obtained in this way is influenced by the state of the vasculature, i.e., vessel rigidity and elasticity, which modulate pulse wave propagation. In young, healthy subjects, RR intervals from ECG, pulse intervals from PPG, and microvascular flow signals may be nearly identical [122]. In older or unhealthy subjects, however, divergence can occur. Simultaneous recordings of respiration, blood pressure, and microvascular flow enable the assessment of oscillatory processes that modulate heart rate and inter-system coupling [19, 20, 120].

### Physiological background

#### Physiological modulation frequencies

Physiological oscillations that modulate the heart rate [19, 114, 123] are:

- *Respiratory oscillations:* Directly linked to respiration; correspond to respiratory sinus arrhythmia [42].
- *Myogenic oscillations:* Generated by vessel wall activity; reflect local vascular smooth muscle dynamics.
- *Neurogenic oscillations:* Arise from sympathetic nervous system activity.
- *Endothelial-related oscillations:* Linked to metabolic and thermoregulatory processes maintained with the involvement of the endothelial cells that are lining the entire cardiovascular system; typically manifest on timescales of minutes.

These oscillations provide important insight into cardiac–respiratory–vascular coupling. In particular, understanding the interactions between the heart and respiratory rhythms is crucial for heart rate variability analysis, assessment of the autonomic nervous system, and the study of cardiovascular or respiratory pathophysiology. This knowledge also opens new avenues for diagnostic tools and therapeutic monitoring using synchronization and coupling metrics (e.g., [45, 121, 134]).

#### Steady versus oscillatory components

HRV consists of *steady (mean)* values and *oscillatory components*, which differ in their response to perturbations:

- *Steady values:* Baseline heart rate or blood pressure can be abruptly altered by posture changes, stress, or drugs.
- *Oscillatory components:* Respiratory, myogenic, neurogenic, and endothelial oscillations have robust phases; amplitude may vary, but phase shifts require coordinated timing.

Methods analyzing HRV must therefore distinguish these components. Stochastic approaches based on statistical descriptions may capture variability but can obscure phase relationships, whereas deterministic or chronotaxic models explicitly represent phase-stable oscillations and their temporal organization.

#### Information provided by HRV

HRV reflects several aspects of cardiorespiratory and vascular regulation:

- *Autonomic regulation:* Early interpretations, based on linear Fourier spectral methods [4, 14, 74], suggested that HRV could provide insight into the balance between sympathetic and parasympathetic activity. However, subsequent studies using wavelet-based frequency analysis, multi-signal cardiovascular measurements, and nonlinear dynamical approaches [23, 29, 35, 120] have shown that the low-frequency (LF, ∼0.04–0.15 Hz) component arises from a mixture of influences, including baroreflex feedback, vascular oscillations, and slower endothelial or metabolic modulation. The high-frequency (HF, ∼0.15–0.4 Hz) component, reflecting respiratory sinus arrhythmia, is predominantly mediated by parasympathetic (vagal) activity [29, 142]. Consequently, the LF/HF ratio is too simplistic to serve as a quantitative measure of sympathovagal balance and should be interpreted with caution [28, 75]. Instead, analyses that account for finite-time dynamics, time-localized stability, and multi-scale interactions provide a more physiologically meaningful characterization of autonomic regulation.
- *Physiological adaptability:* Beyond autonomic tone, HRV captures aspects of physiological adaptability: higher variability generally indicates flexible and resilient physiological regulation, whereas reduced HRV is associated with impaired regulation or dysregulation of physiological systems [132].
- *Coupling across systems:* HRV captures interactions between cardiac, respiratory, and microvascular dynamics [19, 29, 123, 134].
- *Clinical biomarkers:* HRV serves as a clinically relevant biomarker, with reduced variability and altered autonomic regulation associated with cardiovascular disease, diabetes, aging, chronic stress, and increased mortality risk [132, 136].

### Classical analysis

#### Autonomous nonlinear dynamics

Heart rate variability (HRV) reflects fluctuations in the timing of successive heartbeats and corresponds to variability in the instantaneous cardiac frequency.

Within an autonomous dynamical systems description, these fluctuations are interpreted as arising from the intrinsic dynamics of the system, without explicit time-dependent forcing. In this setting, HRV is modeled as the output of a low-dimensional deterministic dynamical system

$$\dot{x}=f(x),$$

where the observed RR-interval series reflects the evolution of an underlying attractor.

Phase-space reconstruction allows the underlying state space to be reconstructed from a single scalar time-series, based on *Takens’ embedding theorem* [130] and related rigorous results by Mañé [77], which guarantee that the dynamics can be unfolded in a sufficiently large embedding dimension.

Analytical tools include:

- *Correlation dimension:* Estimates the effective dimension of the attractor, quantifying the number of active dynamical degrees of freedom [36].
- *Lyapunov exponents:* Measure sensitivity to initial conditions and detect signatures of chaotic dynamics [141].
- *Poincaré maps and return plots:* Geometrically visualize short-term deterministic structure [89, 126].
- *Recurrence analysis:* Recurrence plots and recurrence quantification analysis (RQA) characterize repeated visits of trajectories to similar regions in phase space [78].
- *Nonlinear prediction:* Local phase-space models test whether short-term predictability exceeds that of linear stochastic models [51].

These approaches can uncover deterministic and nonlinear features of HRV that linear spectral methods fail to capture. A key limitation, however, is the assumption of an autonomous system. In reality, HRV arises from interactions among multiple subsystems—including respiration, baroreflexes, vascular tone, neural, endothelial, and metabolic modulation—which both introduce and are influenced by external driving and coupling. As a result, purely autonomous models provide only a partial representation of cardiovascular variability, highlighting the need for nonautonomous dynamical descriptions that explicitly account for time-dependent, adaptive, and context-specific influences.

#### Stochastic dynamics and statistical descriptions

Stochastic dynamics provides a complementary description of complex systems, treating HRV as the output of a *high-dimensional stochastic system*

$$\dot{x} = f(x) + \eta (t),$$

where *f*(*x*) represents deterministic interactions among cardiovascular subsystems, and η(t) captures stochastic influences from unobserved or noisy processes. Unlike low-dimensional nonlinear or linear spectral analyses, this approach emphasizes probabilistic structure, multi-scale correlations, and interactions across subsystems.

Common analytical tools include:

- *Detrended fluctuation analysis (DFA) and multiscale DFA (MFDFA):* Characterize long-range correlations and fractal-like scaling in nonstationary HRV signals [96].
- *Multiscale entropy (MSE):* Evaluates complexity across temporal scales, indicating how predictability or information content changes from short to long timescales [24].
- *Network-based analyses:* Represent HRV time-series as complex networks (e.g., recurrence or visibility graphs) to study time-localized structure, clustering, and connectivity patterns [73].
- *Information-theoretic and causal approaches:* Quantify complexity, predictability, and directional interactions using Shannon entropy, sample entropy, transfer entropy, mutual information, and Granger causality. These methods detect non-trivial couplings and help distinguish true physiological interactions from spurious correlations [31, 72, 90–92, 101].
- *Coupling functions:* Reconstruct the functional relationships between interacting oscillatory systems, capturing directionality, amplitude, and phase influences. Applied to cardiorespiratory signals, they reveal mechanistic interactions beyond statistical correlations [45, 58, 116, 119].

Approaches inspired by statistical physics and complex systems theory capture multi-scale structure, fluctuations, subsystem interactions, and emergent collective behavior, providing a broader description of HRV than classical linear spectral or low-dimensional nonlinear approaches.

**Table 6:** Summary of analytical approaches to HRV, including the assumptions, methods, and principal features captured by each approach

Model	Nonlinear (autonomous) dynamics	Statistical (stochastic) physics	Chronotaxic (non-autonomous)
Assumptions	Deterministic, finite-dimensional	Stochastic, high-dimensional	Driven, time-varying attractor
Analytical methods	Phase-space reconstruction, Lyapunov exponents, attractor analysis	DFA, multiscale entropy, network-based metrics	Time-dependent attractor analysis, wavelet ridge extraction, phase coherence, bispectral methods, coupling functions, order parameters
Key features captured	Intrinsic oscillatory dynamics	Probabilistic structure, multiscale correlations, cross-system interactions	Phase-stable oscillations under physiological forcing, modulatory influences, nonstationary dynamics

However, the nonstationary and non-ergodic nature of HRV can influence the robustness of frequency-domain metrics, scaling exponents, entropy measures, information-theoretic quantities, and network-based metrics. Analyses are sensitive to data segment length and the inherent multi-scale structure of the signal. In particular, simple fixed-length sliding windows are inadequate for capturing dynamics across widely separated time scales, as short windows miss slow components, while long windows smear fast oscillations. Multi-resolution approaches are therefore required. Methods with logarithmic frequency resolution, such as wavelet transforms, provide time-localized representations that adapt naturally across scales, while other multiscale techniques (e.g., entropy-based measures) probe scale-dependent structure without explicitly relying on logarithmic spacing. Careful methodological design and validation—*using surrogate or control data* [66, 108, 133]—remain essential to ensure reliable interpretation.

### Chronotaxic interpretation

As introduced in Sect. 4, chronotaxic systems are characterized by finite-time stability with respect to time-dependent attractors. Within this description, HRV and cardiorespiratory dynamics can be interpreted as outputs of non-autonomous oscillatory systems evolving under continuous physiological modulation

$$\dot{x}=f(x,t), \quad x(t)\ \text {remains close to}\ x_A(t),$$

where $x_A(t)$ denotes the time-dependent reference trajectory associated with the moving attractor generated by ongoing physiological and external influences. Unlike an asymptotic equilibrium or stationary attractor, $x_A(t)$ evolves continuously in time and provides a dynamic reference around which the observed physiological state is organized.

It is important to distinguish between the idealized deterministic trajectory considered in the mathematical formulation of chronotaxicity and the experimentally observed state. Physiological recordings contain contributions from multiple interacting processes that may not be explicitly represented in a reduced model. These influences need not be stochastic; for example, cardiorespiratory coupling, vascular oscillations, and metabolic rhythms can produce systematic frequency modulation and therefore time-dependent deviations from an idealized reference trajectory. Within a chronotaxic interpretation, such deviations are expected to remain bounded, because the dynamics continuously reorganizes around the evolving reference state.

This perspective describes robust oscillatory coordination and transient interactions under non-stationary conditions, extending beyond classical LF/HF analysis and low-dimensional nonlinear measures.

**Fig. 2:**
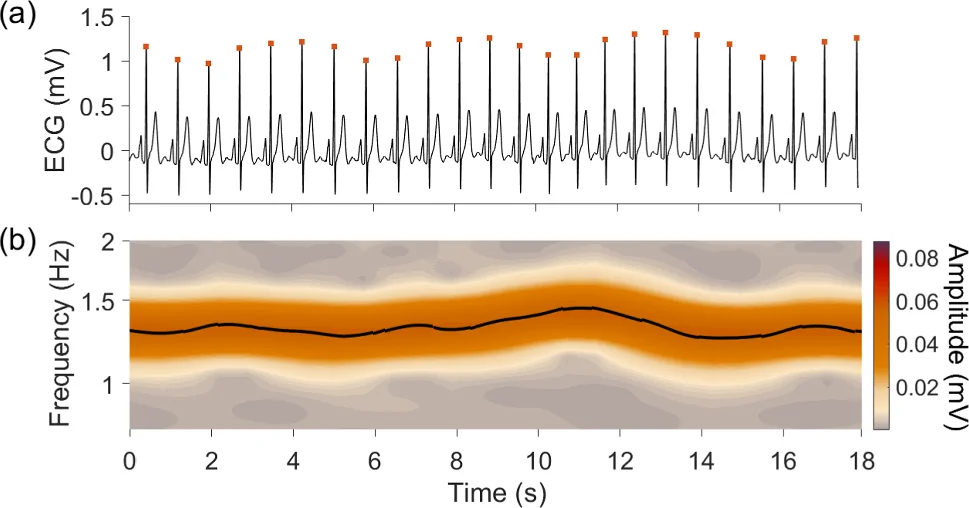
**a** Electrical activity of the heart recorded via ECG, with R-peaks marked in red. **b** Instantaneous heart frequency obtained from ridge extraction of the wavelet transform, with frequency resolution $f_0 = 1.5$ Hz, indicated in black

Analytical approaches include the following:

- *Chronotaxic stability and phase metrics:* The experimental assessment of chronotaxic behavior requires reconstruction of the underlying oscillatory dynamics and analysis of whether interacting components maintain stable relationships, while their frequencies evolve. Phase differences, phase coherence, and frequency coupling provide experimentally accessible measures of dynamical coordination without requiring stationarity or asymptotic synchronization [127].
- *Wavelet-based, time-localized analyses:* Continuous and synchrosqueezed wavelet transforms extract instantaneous amplitude, frequency, and phase from oscillatory signals. HRV can be obtained from suitable physiological signals, including ECG, photoplethysmography (PPG), laser Doppler flowmetry (LDF), or arterial blood pressure, through ridge extraction in the time-frequency plane [48], as illustrated in Fig. 2. Wavelet phase coherence quantifies time-localized phase relationships between oscillatory components, allowing transient interactions to be detected without assuming sustained synchronization [9]. Wavelet bispectral analysis and time-frequency cross-spectral methods provide complementary information about nonlinear interactions and cross-frequency coupling. Multiscale decomposition, including Nonlinear Mode Decomposition (NMD), enables separation of individual oscillatory components prior to analysis, which is particularly important for slow endothelial and metabolic oscillations (∼0.01–0.02 Hz, 50–100 s per cycle) [45, 116].
- *Coupling functions:* Reconstruction of coupling functions provides information about functional relationships between interacting oscillatory subsystems, including the strength, phase dependence, and possible directionality of interactions [116, 119]. In the original chronotaxic formulation, stability of the reference trajectory is associated with a driver–response structure in which the reference dynamics is not altered by perturbations of the response subsystem. In biological systems, where interactions may be reciprocal, coupling analysis provides a means of investigating effective directionality and relative influence between components.
- *Time-dependent order parameters and modulation analysis:* HRV reflects the combined influence of multiple interacting oscillatory processes. The angular-velocity order parameter introduced by Newman et al. [84] quantifies time-dependent coordination of instantaneous frequencies between oscillatory components. In contrast to phase-based measures, it captures alignment of dynamical rates and is therefore suited to systems in which intrinsic frequencies continuously vary. Combined with ridge extraction and multiscale decomposition, such measures enable the investigation of how respiratory, vascular, endothelial, metabolic, and baroreflex oscillations contribute to the evolving organization of HRV.

A complete understanding of HRV requires identification of the relevant modulatory processes and quantification of their contributions over time. Wavelet-based ridge extraction, phase coherence, bispectral analysis, coupling functions, and time-dependent order parameters therefore provide complementary tools for characterizing how multiple physiological oscillators interact within a non-stationary dynamical system.

*Practical considerations:* Slow oscillations require sufficiently long recordings to capture multiple cycles (e.g., ∼30 min for 0.01-−0.02 Hz components). Apparent noise and nonstationarity may reflect deterministic time-dependent dynamics rather than random fluctuations [104]. Analyses should therefore account for preprocessing, respiratory influences, vascular contributions, and multiscale interactions to ensure meaningful interpretation [9, 47, 104].

#### Summary of HRV analytical approaches

HRV can be analyzed using several complementary analytical approaches, each emphasizing specific aspects of cardiovascular and cardiorespiratory dynamics. Table 6 summarizes the core assumptions, analytical methods, and principal features captured by each approach.

Together, these approaches highlight that HRV is inherently *nonstationary, nonergodic, and multiscale*, and that no single method fully characterizes it. Steady-state trends and oscillatory components can behave differently, with abrupt changes in mean values coexisting with phase-robust responses. Peripheral measurements are additionally modulated by vascular properties (rigidity, elasticity, plaques), particularly in older or unhealthy subjects, while respiratory, endothelial, metabolic, and baroreflex oscillations act as physiological modulators.

Accordingly, these analytical approaches should be regarded as *mutually complementary*, with each capturing distinct aspects of HRV dynamics. A comprehensive description therefore requires identification of the relevant modulatory processes together with the use of multiscale, time-resolved methods—including wavelet-based ridge extraction, phase coherence, bispectral analysis, coupling functions, and time-dependent order parameters—to disentangle how individual subsystems shape the observed HRV signal.

**Fig. 3:**
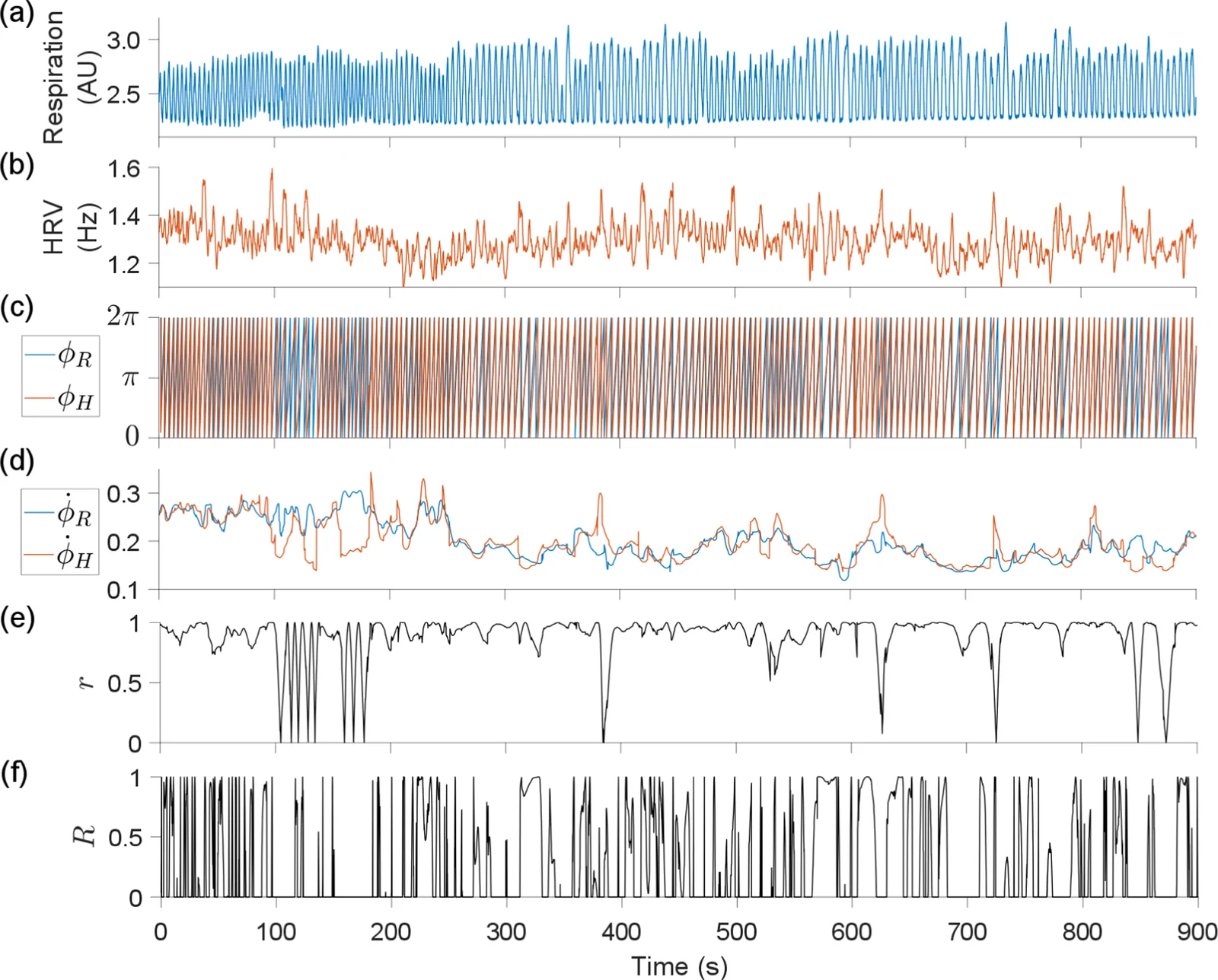
**a** Respiration, **b** HRV, and **c** their instantaneous phases extracted by ridge-based time–frequency analysis using a lognormal wavelet with frequency resolution 1 Hz, **d** instantaneous angular velocities extracted by ridge analysis using a lognormal wavelet with frequency resolution 1.5 Hz, **e** the instantaneous Kuramoto order parameter *r*, and **f** the angular-velocity order parameter *R*. The Kuramoto order parameter remains close to unity for extended periods, with temporal fluctuations. The angular-velocity order parameter exhibits temporal fluctuations in the cardiorespiratory interaction

### Example dataset

Let us now illustrate the chronotaxic approach using simultaneously recorded time-series of cardiac and respiratory activity from a young, healthy subject (number 229 in [125]). A three-lead ECG signal was recorded with electrodes placed on both shoulders and the lowest left rib to maximize R-peak prominence, while an elastic belt with an attached respiratory effort transducer was positioned around the chest to measure respiration. Both signals were recorded at a sampling frequency of 1200 Hz for 30 min, while the subject was relaxed in a supine position at a constant room temperature of 21$^\circ$C.

Traditionally, HRV is obtained from the intervals between consecutive R-peaks [indicated by red dots in Fig. 2a]. An alternative approach is to transform the time-domain data into the time-frequency domain and extract the instantaneous heart rate (defined as instantaneous angular velocity in [84]) as the ridge of the dominant cardiac mode, as described in Sect. 4.5.2 and illustrated in Fig. 2b. Ridge extraction also allows direct access to the instantaneous phase, as shown in Fig. 3c.

The interaction between cardiac and respiratory activity (RSA) arises from respiratory modulation of cardiac frequency and contributes to HRV. In Fig. 3, the respiration signal (a) and the HRV time-series obtained via ridge extraction (b) are shown over a 900 s interval. Instantaneous phases, reconstructed using ridge-based phase extraction with lognormal wavelets and frequency resolution 1 Hz [48], are presented in Fig. 3c, and instantaneous angular velocities, reconstructed using the same method with frequency resolution 1.5 Hz, in Fig. 3d. The corresponding Kuramoto order parameter, introduced in Sect. 4.6, is shown in Fig. 3e, while the angular-velocity order parameter is shown in Fig. 3f.

The Kuramoto order parameter quantifies instantaneous phase coherence, whereas the angular-velocity order parameter reflects time-resolved coordination of the dynamical rates of interacting oscillatory processes. The Kuramoto order parameter indicates extended periods of high cardiorespiratory phase coherence, interrupted by transient reductions associated with intermittent synchronization and phase slips. In contrast, the angular-velocity order parameter reveals that the coordination of cardiac and respiratory frequencies continuously varies over time, even during resting conditions.

This distinction is important for the interpretation of physiological synchronization. A high phase coherence does not necessarily imply stationary coupling, since the underlying frequencies may continue to evolve. The intermittent behavior observed in the angular-velocity order parameter indicates that cardiorespiratory coordination is a dynamic process involving continuous adjustment of instantaneous frequencies rather than a fixed phase-locked relationship.

Within a chronotaxic description, such observations can be interpreted as reflecting coordination around an evolving reference trajectory rather than convergence toward a stationary synchronized state. The respiratory rhythm continuously modulates cardiac dynamics through respiratory sinus arrhythmia, while additional physiological processes contribute further time-dependent modulation. The persistence of coordinated dynamics despite these evolving influences is consistent with a non-autonomous description based on moving attractors and finite-time organization.

This example demonstrates how combining ridge-based phase reconstruction with time-dependent synchronization measures provides an approach for studying physiological coordination beyond stationary approaches. The angular-velocity order parameter reveals time-dependent fluctuations in the coordination of cardiorespiratory dynamics, indicating that synchronization is a continuously evolving process rather than a stationary state.

## From the geometry of biological time to chronotaxic applications

The concept of the geometry of biological time introduced a representation in which physiological dynamics is described as a hierarchy of interacting oscillatory processes spanning multiple time scales [140]. Within this description, biological systems are organized through coordinated rhythms *ranging from slow circadian cycles to fast neural oscillations*, whose interactions shape the variability observed in physiological signals. Chronotaxic dynamics extends this description by providing a mathematical formulation of systems with time-dependent attractors and finite-time stability, enabling a systematic characterization of nonstationary oscillatory coordination in living systems.

In recent years, chronotaxic methods have been increasingly applied to both experimental data and mechanistic modeling of physiological processes spanning multiple biological systems and temporal scales. Chronotaxic analysis enables the identification of time-varying couplings and coordination patterns across physiological subsystems, while chronotaxic modeling represents these interactions using networks of oscillators whose intrinsic properties and couplings evolve in time. In the following sections, we first review experimental studies illustrating the relevance of chronotaxic dynamics for interpreting physiological and pathological data. We then discuss modeling approaches that capture this dynamics using nonautonomous oscillator systems.

### Experimental relevance of chronotaxic dynamics

Methods based on chronotaxic dynamics—including finite-time stability, time-localized phase and amplitude dynamics, and evolving attractor structures—enable the study of nonstationary oscillatory processes in multiscale, non-equilibrium systems [124]. When applied to experimental data or mathematical models, these methods enable the characterization of transient synchrony, adaptive couplings, finite-time coordination, and time-dependent interactions across multiple temporal scales in complex biological and physical systems.

Chronotaxic analysis and related finite-time synchronization methods have been applied in a variety of experimental contexts. Diagnostic classification of skin melanocytic lesions has been performed using blood perfusion fluctuations [64]. In humans, time-frequency analysis of heart rate variability, phase coherence, and coupling functions revealed multiscale autonomic regulation beyond the baroreflex [23], while coherence and coupling analyses identified microvascular impairment, demonstrating sensitivity to subtle dysfunctions in vascular dynamics [134]. Time-localized phase and oscillatory analysis methods have also been used to characterize oscillations of human subarachnoid space width in the resting state, revealing multiscale coordination in cerebrospinal dynamics [37].

Cardiorespiratory phase coherence during hypoxia has been linked to genetic polymorphisms, highlighting the sensitivity of phase coherence to individual physiological variability [67], and phase coherence between cardiovascular oscillations in malaria provides a potential basis for diagnostic assessment [2].

In animal models, mice lacking Per1/2/3 clock genes exhibited residual circadian-like rhythms, highlighting the effects of genetic perturbation on the circadian oscillator [80], and pharmacological modulation with methamphetamine further altered the oscillator and its multiscale couplings [10].

Time-localized analysis also revealed developmental and pathological alterations in neural connectivity: theta and alpha connectivity patterns were modulated in children with autism spectrum disorder [11].

Cortico-muscular phase connectivity during motor tasks has further demonstrated the relevance of phase-based interaction measures in clinical populations, including early Parkinson’s disease [88], while a related study highlighted the role of phase dynamics in motor and cognitive coordination [76].

Neurovascular phase coherence was found to be disrupted in Alzheimer’s disease [18] and Huntington’s disease [17], while aging altered spontaneous phase coherence between neural activity and brain oxygenation [16]. Furthermore, alterations in the coupling functions between cortical and cardiorespiratory oscillations due to anesthesia with propofol and sevoflurane were captured using chronotaxic analysis, demonstrating sensitivity to physiological state transitions [118].

Together, these studies illustrate that time-varying oscillations can be quantified and tracked to reveal behavior and interactions across physiological systems, temporal scales, and perturbations. This approach provides a finite-time, non-ergodic characterization of complex biological oscillations that is directly measurable in experiments.

### Modeling with chronotaxic dynamics

Chronotaxic dynamics provides a mathematical approach for modeling biological systems in which oscillatory processes exhibit finite-time stability and time-dependent attractor structures. In the context of cellular and brain metabolism, this approach has been progressively developed over several studies. Early work modeled cell energy metabolism as networks of non-autonomous oscillators, using weighted interactions to capture cross-system couplings and identify altered metabolic states [65, 103]. These studies demonstrated that non-autonomous phase oscillators could capture transient phase relationships, multiscale coordination, and context-dependent interactions in metabolic networks.

A related application to cardiorespiratory dynamics further demonstrated the utility of this modeling approach for physiological oscillator networks. Brimble, Clemson, and Stefanovska [21] modeled cardiorespiratory interactions as coupled non-autonomous oscillators with time-varying frequency modulation, showing that observed synchronization patterns arise from the ability of the system to track evolving attractors rather than from stationary phase-locking conditions.

Building on this foundation, this modeling approach was extended to neuronal metabolism [12], representing each metabolic subprocess as a phase oscillator with time-varying intrinsic frequency and coupling. The system is described by equations of the form

$$\dot{\phi }_i(t) = \omega _i(t) + \sum _{j\ne i} K_{ij}(t) H(\phi _j(t) - \phi _i(t)),$$

where $\phi _i(t)$ are oscillator phases, $\omega _i(t)$ are time-dependent frequencies, $K_{ij}(t)$ are dynamic coupling strengths, and *H* is a coupling function specifying how phase differences influence interactions. The explicit time dependence in $\omega _i$ and $K_{ij}$ generates evolving attractor structures, allowing the model to capture the drifting synchrony, transient phase-locking, and scale-dependent coordination observed in real metabolic systems. The use of non-autonomous oscillators here reflects the defining requirement of chronotaxicity that the underlying attracting structure itself evolves in time.

Unlike classical autonomous models or stationary spectral analyses, non-autonomous phase oscillator models provide a finite-time, non-ergodic description of complex metabolic dynamics. By reconstructing time-dependent frequencies, phase relationships, and coordination patterns from experimental data, these models reveal interpretable features of multiscale interactions, cross-system couplings, and responses to perturbations. This chronotaxic modeling approach therefore enables a detailed mechanistic description of biological rhythms including cellular, cardiorespiratory, and brain metabolic dynamics, linking mathematical theory with experimentally observable properties and supporting investigation of altered metabolic states in health and disease.

## Summary

Taken together, classical dynamical systems theory and stochastic approaches developed within statistical physics provide a powerful and internally consistent description of physical and biological systems in terms of asymptotic stability, invariant measures, and long-time statistical regularity. However, in their classical formulations, these descriptions are largely based on structures defined through long-time limits, invariant sets, or stationary statistical properties. Consequently, temporal variability is often interpreted in relation to these underlying structures, for example as transient behavior, fluctuations, or deviations from statistically regular states.

In living systems, by contrast, variability is not merely a perturbation around an underlying fixed structure but can itself participate in functional organization. Physiological and neural processes continuously adapt to changing internal and external conditions while preserving coordinated function, implying that the relevant coordination structures may themselves evolve in time. This introduces a conceptual mismatch between asymptotic invariants and experimentally observed finite-time coordination, motivating descriptions in which stability and organization are defined relative to time-dependent structures rather than time-independent attractors.

Chronotaxicity offers a formal description of a class of non-autonomous dynamical systems relevant to living systems, viewed through their dynamic, adaptive, resilient, and coordinated temporal organization, thereby capturing their *organized non-equilibrium* nature. Rather than converging to fixed points or stationary long-term attractors, biological systems may be treated as maintaining function through temporally structured interactions that span multiple scales. This formulation emphasizes that coordinated timing can emerge through interactions between oscillatory processes whose phases and frequencies continuously adjust, thereby sustaining adaptability, robustness, and persistent functionality across scales.

By integrating insights from phase coordination and non-autonomous dynamics, chronotaxicity offers a conceptual and mathematical basis for investigating the temporal organization of living systems and suggests experimental approaches to probe these principles across physiology, neuroscience, systems biology, and ecology. More broadly, this perspective points toward a physics of living systems that recognizes stability and function as emergent properties of continuously maintained temporal coordination rather than solely of static asymptotic states.

## Outlook

The recent experimental demonstration of chronotaxicity in a physical system [115] provides an opportunity to extend chronotaxic analysis beyond its original biological applications and to investigate its predictive potential in controlled experimental settings. Future work should focus on quantitative characterization of coordination in non-stationary systems using finite-time measures, including time-localized phase coherence [9, 112] and angular-velocity alignment [84]. Combined with mode decomposition methods [46, 107], recent developments in non-autonomous dynamical systems [83], and finite-time stability theory under slowly varying forcing [82], these approaches offer routes toward quantitative descriptions of adaptive, multiscale oscillatory dynamics. Toolboxes such as MODA (Multiscale Oscillatory Data Analysis; https://github.com/luphysics/MODA) facilitate the reproducible implementation of these methods.

An important theoretical challenge is extending chronotaxicity from individual oscillators to interacting networks with continuously varying frequencies and adaptive coupling. Building upon methods for reconstructing time-dependent coupling functions in oscillator networks [117], future developments may enable the identification of chronotaxic clusters, characterization of perturbation propagation, and analysis of transiently stable modules in high-dimensional adaptive systems.

Another important direction for future research is the further development of phase theory for non-autonomous systems. While time-localized methods such as ridge extraction [48] and nonlinear mode decomposition [46] provide practical means of reconstructing instantaneous phase and frequency from evolving oscillations, and a mathematical formulation of phase for non-autonomous oscillatory systems has recently been proposed [83], an important next step is to establish a closer correspondence between the mathematical definition of phase and its reconstruction from experimental data. Such a development would enable quantities derived from phase, such as instantaneous angular velocity [84], to be extracted with a clear connection to their underlying mathematical definition, thereby improving the interpretation of frequency-based synchronization measures and facilitating the investigation of chronotaxicity. More broadly, this would provide a basis for analyzing synchronization, coupling, and temporal organization in living systems.

The broader significance of chronotaxicity extends beyond physiology to other systems characterized by coupled, non-stationary oscillations. Potential applications include ecology, engineering, data science, economics, and nonlinear physics, where evolving temporal organization may play an important functional role. Whether chronotaxicity represents a general property of adaptive non-autonomous systems across these domains remains an open question that will require further theoretical development, quantitative methodology, and experimental validation.

More generally, the developments reviewed here highlight temporal organization as a fundamental aspect of living systems. By providing a mathematical description of stability and coordination in evolving dynamical environments, chronotaxicity contributes to the broader effort to develop a physics-based understanding of life, in which function and resilience emerge from continuously maintained temporal relationships rather than solely from stationary asymptotic states.

## Data Availability

Datasets for the participant included in the study are available on PURE (https://doi.org/10.17635/lancaster/researchdata/747), as listed in Ref [125].
